# What are the Implications for Policy Makers? A Systematic Review of the Cost-Effectiveness of Screening and Brief Interventions for Alcohol Misuse in Primary Care

**DOI:** 10.3389/fpsyt.2014.00114

**Published:** 2014-09-01

**Authors:** Colin Angus, Nicholas Latimer, Louise Preston, Jessica Li, Robin Purshouse

**Affiliations:** ^1^School of Health and Related Research (ScHARR), University of Sheffield, Sheffield, UK; ^2^Department of Automatic Control and Systems Engineering, University of Sheffield, Sheffield, UK

**Keywords:** alcohol drinking, screening and brief intervention, primary care, systematic review, policy making, resource allocation, brief alcohol intervention, brief intervention

## Abstract

**Introduction:** The efficacy of screening and brief interventions (SBIs) for excessive alcohol use in primary care is well established; however, evidence on their cost-effectiveness is limited. A small number of previous reviews have concluded that SBI programs are likely to be cost-effective but these results are equivocal and important questions around the cost-effectiveness implications of key policy decisions such as staffing choices for delivery of SBIs and the intervention duration remain unanswered.

**Methods:** Studies reporting both the costs and a measure of health outcomes of programs combining SBIs in primary care were identified by searching MEDLINE, EMBASE, Econlit, the Cochrane Library Database (including NHS EED), CINAHL, PsycINFO, Assia and the Social Science Citation Index, and Science Citation Index via Web of Knowledge. Included studies have been stratified both by delivery staff and intervention duration and assessed for quality using the Drummond checklist for economic evaluations.

**Results:** The search yielded a total of 23 papers reporting the results of 22 distinct studies. There was significant heterogeneity in methods and outcome measures between studies; however, almost all studies reported SBI programs to be cost-effective. There was no clear evidence that either the duration of the intervention or the delivery staff used had a substantial impact on this result.

**Conclusion:** This review provides strong evidence that SBI programs in primary care are a cost-effective option for tackling alcohol misuse.

## Introduction

The misuse of alcohol is a substantial concern for public health policy makers across the world, with over 5% of the global burden of disease and injury estimated as being alcohol-attributable (1). In addition to these deleterious effects on health and the associated economic costs, excessive consumption of alcohol is also associated with a range of social harms such as increased crime, public nuisance, and reduced workplace productivity, which impact not just on the drinker, but on society as a whole (2).

Primary care provides an avenue through which a large proportion of the population can be reached by interventions aimed at reducing alcohol misuse and the related consequences. In particular, excessive drinkers attend primary care with greater frequency than moderate drinkers (3) and may therefore be more easily targeted through this channel. Programs of Screening and Brief Interventions (SBIs), in which patients are screened opportunistically for alcohol misuse and those screening positively are offered a brief session of advice can harness these properties to achieve broad coverage of the population at risk (4).

There is a substantial body of existing research into the effectiveness of SBI programs in primary care, with a recent review of reviews identifying 24 previous systematic reviews (5). The consistent finding of these studies is that SBIs are effective at reducing excessive alcohol consumption and this weight of evidence has led to the inclusion of SBIs in a range of international policy recommendations including the World Health Organisation’s global strategy for tackling harmful alcohol use (6). However, in spite of these calls for the implementation of such policies, evidence on the cost-effectiveness of SBI programs is less equivocal. This is a key question for the policy makers and healthcare budget planners being urged to instigate or fund these programs and there have been few attempts to draw together the existing literature in order to inform their decisions.

There have been three major previous reviews of the cost-effectiveness evidence on SBIs in primary care (7–9). While all three conclude that they are cost-effective, none examine the impact that implementation decisions such as the staff used to deliver the SBI, or the duration of the intervention itself, have on overall program cost-effectiveness. These issues are critical as the use of general practitioners (GPs) to deliver SBIs is usually a substantially more expensive option than nursing staff and a lack of available time is the single greatest perceived barrier to early intervention in alcohol problems in primary care (10). In addition, these existing reviews either predate several important studies or have a narrow scope which misses a number of key papers. This study updates and expands the 2008 review by Latimer et al. (8) in order to provide a systematic overview of the existing cost-effectiveness evidence for SBIs in primary care, together with an examination of the differential impact of alternative implementation options.

## Methods

The original search was undertaken in May 2008 (8) and refreshed on four subsequent occasions, with the latest update undertaken in April 2014. Searches were conducted on the following electronic databases:

Medline in Process and Other Non-Indexed Citations and Medline 1950-present via OVID SP
EMBASE via OVID SP
Science Citation Index via Web of Knowledge
Social Science Citation Index via Web of Knowledge
Cochrane Library Databases via Wiley
Assia via CSA
PsycINFO via OVID SP
Econlit via OVID SP

**Figure 1:**
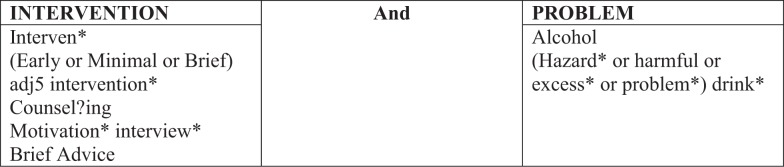
**Search strategy utilized in the review**.

The original search undertaken in 2008 adopted an iterative emergent approach. Rather than developing an *a priori* search strategy, smaller individual searches were undertaken in order to develop understanding of the research area. The information specialist (Louise Preston) and lead reviewer (Nicholas Latimer) worked together to develop further iterations of the search strategy based on the findings of earlier searches. As a result, for this update, the use of a predetermined search strategy was possible as search terms had been tested and validated as part of the original searches. The search strategy is presented in Figure 1.

The title and abstracts of all retrieved studies were screened by one reviewer (Colin Angus) against a set of pre-defined inclusion and exclusion criteria. These criteria, listed in Supplementary Material, were piloted with a second reviewer (Jessica Li) on an initial subsample of 10 studies and subsequently refined, following discussions between both reviewers, to ensure clarity in their interpretation. Any study reporting the costs and health or other economic benefits of SBI programs in primary care were considered for inclusion. Studies were excluded which were not published in English, which examined multi-behavior interventions (e.g., combined drink and drugs education programs), which included components occurring outside of primary care, or which evaluated interventions comprising more than four patient contacts (on the grounds that these no longer constitute “brief” interventions). Studies examining SBI implementation strategies only (e.g., GP education programs to increase delivery rates of SBIs to patients) were excluded unless they presented a separate economic evaluation of the SBI delivery itself. Similarly, studies that examined only screening tools (e.g., AUDIT or CAGE) were excluded unless they also included a BI component.

Data from all included studies were extracted by one reviewer (Colin Angus) using a standard template (see Supplementary Material) adapted from that used by Latimer et al. (8). Studies were assessed for methodological quality using the Drummond checklist for economic evaluations (11) as recommended for use in Cochrane reviews (12). Five of the included studies were randomly selected and additionally assessed for quality by a second reviewer (Jessica Li) to ensure consistency (agreement was 100% between both reviewers).

## Results

Twenty-three papers reporting the results of 22 distinct studies that met the criteria for inclusion in the review were identified. These fall into two major categories: economic evaluations alongside clinical trials (EEACTs) (13–21) and stand-alone modeling evaluations (4, 7, 22–33). Table 1 summarizes these studies, while excluded studies are reported in Supplementary Material. A glossary of relevant health economic terms is included in Supplementary Material.

**Table 1:** **Characteristics of included studies**.

Study	Country	Study type	Comparators	Costs included	Health outcomes included	Results	Quality	Duration of intervention	BI delivery staff
Angus et al. (28)	Italy	CUA	(1) Do-nothing scenario (2) Screening with AUDIT-C followed by 10 min brief intervention	Intervention costs and health and social care resource use over 30 years following start of program	QALYs gained over 30 years follow-up	SBI delivered at next GP registration has an ICER of €550 per QALY vs. do-nothing. SBI at next GP consultation has an ICER of €590 per QALY vs. do-nothing.	++	10 min	GP
Angus et al. (33)	Netherlands and Poland	CUA	(1) Do-nothing scenario (2) Screening with AUDIT-C followed by 10 min brief intervention	Intervention costs and health and social care resource use over 30 years following start of program	QALYs gained over 30 years follow-up	Netherlands: SBI delivered at next GP registration has an ICER of €6340 per QALY vs. do-nothing. SBI at next GP consultation has an ICER of €5748 per QALY vs. do-nothing. Poland: SBI delivered at next GP registration has an ICER of zł3696 per QALY vs. do-nothing. SBI at next GP consultation has an ICER of zł3269 per QALY vs. do-nothing.	++	10 min	GP
Babor et al. (15)	USA	EEACT/CEA	Screening with AUDIT followed by either: (1) Treatment as usual (2) 3–5 min brief intervention	Intervention costs	SF-12 score and mean alcohol consumption at 12 months follow-up	Small but significant reduction in consumption for BI group vs. treatment as usual. No significant difference in SF-12 scores. No significant differences in either outcome between GP- and nurse-delivered intervention groups	−	3–5 min	GP or nurse
Chisholm et al. (30)	International	CUA	(1) Do-nothing scenario (2) Screening followed by brief intervention involving four primary care visits inside a year	Intervention costs	DALYs averted over a lifetime horizon	SBI varies from dominated by to dominating a do-nothing scenario depending on WHO region with 9/12 regions having an ICER of ≤5000I$ per QALY	+	Not stated	GP
Cobiac et al. (22)	Australia	CUA	(1) Do-nothing scenario (2) Screening followed by counseling, supportive written materials and follow-up consultations with further advice “if necessary”	Intervention costs, patient time/travel and health and social care resource use over lifetime horizon	DALYs averted over a lifetime horizon	ICER of $6800 per DALY averted vs. do-nothing	−	Not stated	GP
Dillie et al. (16)	USA	EEACT/Cost minimization analysis	Screening with self-reported alcohol consumption followed by either: (1) 2 × 15 min brief interventions each followed up with a 5 min telephone call (2) Additional screened with % CDT followed by 2 × 15 min brief interventions each followed up with a 5 min telephone call	Intervention costs, patient time/travel, health and social care resource use, motor vehicle crashes and legal/criminal costs over 4 years follow-up	N/A	Addition of % CDT screening saves $212 per patient screened	+	40 min	GP (nurse delivers follow-up phone calls)
Drummond et al. (14)	UK (Wales)	EEACT/CUA	Screening with AUDIT followed by either: (1) 5-min nurse-led “minimal intervention” (2) ”Stepped care” – 20 min behavioral change counseling session followed up with referral to motivational enhancement therapy and/or specialist alcohol services if indicated	Intervention costs, health and social care resource use costs and costs of crime at 6 months follow-up	QALYs gained at 6 months follow up	Stepped care 98% likely to be most cost-effective option at a threshold of £20,000–30,000 per QALY. No ICER presented	−	5 min (minimal intervention) or 20+ min (stepped care)	Practice nurse
Fleming et al. (17, 18)	USA	EEACT/CBA	Screening with 7-day timeline follow back followed by either: (1) Patient information leaflet (2) 2 × 15 min brief interventions each followed up with a 5 min telephone call	Intervention costs, patient time/travel, health and social care resource use, motor vehicle crashes and legal/criminal costs over lifetime horizon	Mean alcohol consumption at various points up to 4 years follow-up	Significant reduction in consumption observed in SBI group (32% in men, 43% in women). SBI estimated to save $546 per patient from healthcare perspective and $7780 from a societal perspective vs. patient information leaflet	+	40 min	GP (nurse delivers follow-up phone calls)
Freeborn et al. (19)	USA	EEACT/Resource utilization analysis	Screening with AUDIT followed by either: (1) Treatment as usual (2) Brief advice from GP then 15 min motivational session with trained counselor	Health and social care resource use over 2 years follow-up	N/A	No significant difference in health and social care resource use between BI and care as usual groups	−	15+ min	GP and trained counselor
Freemantle et al. (29)	International	CEA	(1) Do-nothing scenario (2) Screening with AUDIT followed by 15 min brief intervention	Intervention costs	Mean alcohol consumption at 24 months follow-up	SBI costs £8–20 per patient, which equates to £18–47 per patient who reduces their drinking, with a mean reduction of 24% among those who cut down	−	15 min	GP
Kapoor et al. (23)	USA	CUA	(1) Do-nothing scenario (2) Screening with AUDIT followed by full clinical assessment of unhealthy alcohol use and 5–10 min brief intervention (3) Screening with AUDIT and % CDT followed by full clinical assessment of unhealthy alcohol use and 5–10 min brief intervention	Intervention costs, health and social care resource use over lifetime horizon	QALYs gained over lifetime horizon	Both screening strategies dominate vs. do-nothing. Incremental cost of adding % CDT to screening is $15,500 per QALY	+	5–10 min	Not stated
Lock et al. (20)	UK (England)	EEACT/Cost minimization analysis	Screening with AUDIT followed by either: (1) Treatment as usual (2) 5–10 min nurse-led brief intervention	Intervention costs, health and social care resource use and personal costs at 12 months follow-up	SF-12 score at 12 months follow-up	No statistically significant difference in costs or health outcomes between arms	+	5–10 min	Nurse
Ludbrook et al. (7)	UK (Scotland)	CEA	(1) Do-nothing scenario (2) Screening using 7-day timeline follow back followed by 2 × 15 min brief interventions each followed up with a 5 min telephone call	Intervention costs, patient time/travel, health and social care resource use, motor vehicle crashes and legal/criminal costs over lifetime horizon	Life years gained over lifetime horizon	SBI dominates vs. do-nothing	−	40 min	GP (nurse delivers follow-up phone calls)
Mundt et al. (21)	USA	EEACT/CBA	Screening with health screening survey and assessment interview followed by either: (1) Treatment as usual (2) 2 × 15 min bried interventions each followed up with a 5 min telephone call	Intervention costs, patient time/travel and health and social care resource use over 2 years follow-up	Life years lost (valued at $50,000 each) over 2 years follow-up	Non-significant cost savings of $467 from healthcare perspective and $812 from societal perspective for BI vs. treatment as usual	+	40 min	GP (nurse delivers follow-up phone calls)
Navarro et al. (24)	Australia	CEA	(1) Current level of SBI provision (2) Increased levels of screening and brief intervention or combined SBI provision	Intervention costs (including training)	Number of risky drinkers who reduce their alcohol consumption	Additional cost of between $174–1041 per risky drinker who reduces their drinking, depending on the scenario	+	Not stated	GP
Purshouse et al. (4)	UK (England)	CUA	(1) Do-nothing scenario (2) Screening with AUDIT followed by 5 min brief intervention	Intervention costs and health and social care resource use over 30 years following start of program	QALYs gained over 30 years follow-up	SBI delivered at next GP registration dominates do-nothing scenario. SBI at next GP consultation has an ICER of £1175 per QALY vs. do-nothing	++	5 min	Practice nurse/GP (both modeled)
Rehm et al. (27)	Canada	CBA	(1) Do-nothing scenario (2) Screening followed by brief intervention	Health and social care resource use costs, costs of crime and productivity losses due to death and disability per annum. Unclear if intervention costs are included	Deaths, years of life lost and acute hospital days averted per annum	Introduction of BI would avoid 360 deaths, 9000 years of life lost, 56,000 acute care hospital days and would reduce alcohol-attributable costs by $602m per annum vs. do-nothing	+	Not stated	Not stated
Saitz et al. (31)	USA	CUA	(1) Do-nothing scenario (2) Screening followed by brief intervention	Intervention costs and health and social care resource use over lifetime horizon	QALYs gained over a lifetime horizon	SBI dominates vs. do-nothing	−	Not stated	Not stated
Solberg et al. (25)	USA	CUA	(1) Do-nothing scenario (2) Annual screening followed by 5 min BI	Intervention costs, patient time/travel and health and social care resource use over lifetime horizon	QALYs gained over lifetime horizon	ICER of $1750 per QALY vs. do-nothing with healthcare perspective. SBI dominates with societal perspective	+	5 min	GP
Tariq et al. (26)	Netherlands	CUA	(1) Do-nothing scenario (2) Screening with AUDIT followed by 10–15 min brief intervention	Intervention costs and health and social care resource use costs over a lifetime horizon	QALYs gained over lifetime horizon	ICER of €5400 per QALY gained for brief interventions vs. do-nothing	++	30–45 min	GP
Watson et al. (13)	UK (England and Scotland)	EEACT/CUA	Screening with AUDIT followed by either: (1) 5-min nurse-led “minimal intervention” (2) “Stepped care” – 20 min behavioral change counseling session followed up with referral to motivational enhancement therapy and/or specialist alcohol services if indicated	Intervention costs and health and social care resource use at 6 and 12 months follow-up	QALYs gained at 6 and 12 months follow-up	ICER of £1100 per QALY for stepped gain over minimal intervention at 6 months, stepped care dominates at 12 months	++	5 min (minimal intervention) or 20+ min (stepped care)	Practice nurse
Wutzke et al. (32)	Australia	CEA	(1) Do-nothing scenario (2) Screening with AUDIT followed by 5 min brief intervention	Intervention costs (including training and support for GPs)	Life years gained (time horizon not stated)	ICER of between $586–650 per life year gained for SBI vs. do-nothing	+	5 min	GP

*CBA, cost–benefit analysis; CDT, carbohydrate deficient transferrin; CEA, cost-effectiveness analysis; CUA, cost-utility analysis; DALY, disability-adjusted life year; EEACT, economic evaluation alongside a controlled trial; GP, general practitioner; ICER, incremental cost–effectiveness ratio; N/A, not applicable; QALY, quality-adjusted life year; SBI, screening and brief interventions. For detailed definitions of terms see Supplementary Material*.

These 23 studies examine the cost-effectiveness of SBIs in almost exclusively high-income countries (Chisholm et al. being the only exception (30)), with the majority of studies covering the USA (8 studies), UK (5 studies), or Australia (3 studies). There was considerable variation in the quality of the studies, with 7 rated as being of low quality, 10 of moderate quality, and 5 of high quality, although there are signs of an improving trend over time with more recent papers scoring more highly. The main issues encountered were an inadequate description of the intervention itself, poor reporting of the sources of cost data used in the studies, and insufficient sensitivity analysis.

Of the nine studies reporting evaluations alongside clinical trials, two compared different levels of brief intervention (13, 14), both concluding that a longer “stepped care” intervention was the most cost-effective option. Another six studies compared brief interventions with usual care (15, 17–21). The trials that these studies are associated with ran for between 6 and 48 months, while the full effect of changes in drinking behavior on health outcomes can take many years to develop (34). It is therefore perhaps unsurprising that these studies found few statistically significant results and do not allow any firm conclusions to be drawn around the cost-effectiveness of SBI programs.

All except one of the 14 modeling studies compared SBI provision to an alternative do-nothing scenario in which no SBIs are delivered. The other study (24) examined the cost-effectiveness of increasing the current uptake rate. Among these studies, the most common health outcome measures were QALYs (4, 23, 25, 26, 28, 31, 33), with two studies using DALYs (22, 30) and two using life years gained (7, 32). Almost all these studies found SBIs to be either cost-saving and health improving (i.e., they dominate a do-nothing scenario) or to have very low costs relative to health gains, making SBI programs highly likely to be considered cost-effective under the relevant national guidelines. The sole exception was Chisholm et al. (30), who presented separate costs and benefits for each of 12 World Health Organization (WHO) sub-regions and found that SBI programs are dominated by current taxation in parts of Africa (region AfrE), although they estimated that they are either cost-effective or cost-saving in the remaining 11 sub-regions using the WHO’s estimated cost-effectiveness thresholds (35). Of the remaining modeling studies, one (27) uses a burden of disease approach to estimate SBIs would be substantially cost-saving (Canadian $602m per annum). The remaining studies (24, 29) use intermediate end points (number of risky drinkers averted and change in mean alcohol consumption), which make the results unhelpful for the purpose of informing resource allocation decisions without additional modeling to estimate the impact of these end points on health outcomes. The majority of these modeling studies consider outcomes over a 30 year (4, 28, 33) or lifetime (7, 22, 23, 25, 26, 30, 31) time horizon, ensuring that the long-term impacts are reflected in the results.

Fifteen studies examine the cost-effectiveness of GP-delivered interventions (4, 7, 15, 16, 18, 21, 22, 24–26, 28–30, 32, 33), while only five examine nurse-delivered interventions (4, 13–15, 20). Owing to the substantial heterogeneity between studies both in terms of methods and outcomes it is difficult to draw any clear conclusions about the relative cost-effectiveness of using different staff to deliver SBI programs, although the lack of a clear difference between the two options may be of interest to policy makers. Only two studies directly compare both options: Purshouse et al. (4) assume *a priori* that delivery staff do not impact on the effectiveness of the BI but find that even the use of the more expensive GP-delivered BI option is unlikely to prevent the program from being cost-effective. Meanwhile, Babor et al. (15) conducted a trial with separate nurse-delivered and GP-delivered SBI arms. The authors found no significant difference in effectiveness of the intervention between these arms, while the nurse-delivered option was around 1/3 cheaper, indicating it to be a more cost-effective option.

With regards to the total duration of the intervention (i.e., the total contact time between patient and delivery staff, either face-to-face or over the telephone, aggregated over multiple contacts where appropriate), 12 studies evaluate interventions of 10 min or less (4, 13–15, 20, 23, 25, 28, 32, 33) and 11 consider interventions of over 10 min (with a maximum duration of 45 min) (4, 7, 13, 14, 16, 18, 21, 26, 28, 29). Again the heterogeneity of methods and outcomes makes direct comparison difficult, although there is no clear difference in terms of cost-effectiveness between shorter and longer interventions. Only five studies consider both longer and shorter interventions. Two of these (13, 14) report that the longer intervention is cost-effective relative to the shorter one, although this conclusion is difficult to make on the basis of the analysis presented in the studies, particularly given the short follow-up of the trials. The other three studies (4, 28, 33) assume no difference in effectiveness but find that longer, more expensive interventions are still highly likely to be considered cost-effective compared to no intervention.

**Figure 2:**
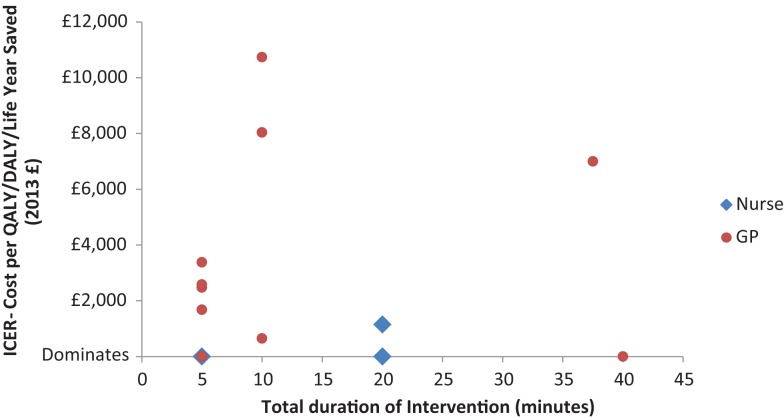
**Cost-effectiveness of SBI programs by SBI duration and delivery staff**.

In order to further explore the relationship between delivery staff, BI duration, and cost-effectiveness, Figure 2 presents a direct comparison of the cost-effectiveness results converted to 2013 UK £, for those studies which report delivery staff, intervention duration, and an Incremental Cost–Effectiveness Ratio (ICER) (4, 13, 25, 26, 28, 33, 36).

## Discussion

This systematic review provides strong evidence that SBIs in a primary care setting are a cost-effective policy option for tackling alcohol-related harms, at least in high-income countries. There is a paucity of evidence for lower- or middle-income countries and that does exist indicates that there may be substantial heterogeneity in both the expected costs and effectiveness of SBI programs depending on the local context in these areas (30).

There is also substantial heterogeneity in study methods, included costs, and reported health outcomes between the included studies, which makes it difficult to determine the implications of this diverse body of evidence for those making resource allocation decisions, although there is an apparent trend for more recent studies to use standardized measures such as QALYs or DALYs, which makes between-study comparison more meaningful. There are also significant differences in the national contexts between studies (for example the existing level of drinking or the current suite of alcohol policies in the country), which must be considered when making international comparisons.

Considering these differences, there is no clear evidence that the choice of delivery staff for SBI programs has a substantial impact on the program’s cost-effectiveness. This may be because GP-delivered interventions are more effective but more costly than those delivered by nurses, although this would be at odds with existing literature, which suggests that the use of less costly nursing staff to conduct tasks that would otherwise be the responsibility of GPs is unlikely to impact negatively on the quality of care received by patients (37, 38). Figure 2 also suggests that nurses may be a more cost-effective option, although heterogeneity in settings and methods between the included studies mean that the graph should be interpreted with caution.

It is also important to note that policy makers will need to consider the total budget impact of any policy options in addition to the potential cost-effectiveness, an issue highlighted in several of the included studies (28, 33, 39). This may suggest that nurse-led SBI programs, which are likely to be less costly overall, may be more appealing option, although consideration must be given to the existing primary care systems in each country. For example, in countries such as the UK or the Netherlands where practice nurses already undertake many primary care services such as vaccinations or health checks, nurse-led SBIs may be a more practical option than in other countries where care is currently delivered exclusively by the GP.

There is also no clear evidence that the duration of intervention delivered has a substantial impact on cost-effectiveness. Again this may indicate that longer interventions are more effective but more expensive, although studies on the effectiveness evidence have not found a consistent relationship between amount of patient contact and effectiveness (5, 40). While the studies by Watson and Drummond provide limited evidence that longer interventions may be more cost-effective in the short-term in the UK context, it is not clear that this translates to the longer term, or to other countries (13, 14).

In addition to the substantial heterogeneity between studies already mentioned, there are a number of limitations to this systematic review. Only studies published in the English language were included, something which may be at least partly responsible for the lack of included studies from the developing world. Some of the included studies are also of low methodological quality which makes it difficult to evaluate the robustness of their conclusions. Finally, there are two key issues, which no study of SBI effectiveness can escape. The first is that the estimates of effectiveness, which underpin the cost-effectiveness estimates examined here may be exaggerated by the impact of regression to the mean, caused by drinkers changing their consumption over time for reasons unrelated to the receipt of a brief intervention (e.g., public holidays or seasonal variation) (41). The second, countervailing issue is that of an intervention or Hawthorne effect, whereby the act of being enrolled into a trial acts as an intervention in itself, something which may at least partly explain why many SBI effectiveness studies observe a reduction in alcohol consumption over time in the control groups (42).

Limitations in the evidence base mean that this review is unable to address a number of other issues that may be of interest to policy makers such as the cost-effectiveness of SBI programs targeting specific groups within the general population. Further research to examine the differential effectiveness of, and the likely coverage by, SBI programs in these subgroups is important to allow this area to be explored further. The other key priority for further research to inform decision makers concerns the uptake among primary care providers of SBI programs. Difficulties in persuading GPs and nurses to fully deliver SBI programs could have a substantial impact on the effectiveness and cost-effectiveness of these programs. A recent international trial conducted as part of the optimizing delivery of healthcare interventions (ODHIN) project will go some way to addressing this challenge by examining the effectiveness and cost-effectiveness of different strategies at increasing SBI delivery rates in primary care (43).

In conclusion, while there are significant differences between the studies included in this review, the overwhelming conclusion is that SBIs in primary care are a cost-effective option, at least in high-income countries. There is no clear evidence that the duration of the intervention, or the type of staff used to deliver it, changes this conclusion. Policy makers should, however, be mindful of the differing budget implications that alternative implementation options may present.

## Author Contributions

Louise Preston undertook the literature searches. Colin Angus conducted the review, with assistance from Jessica Li, and drafted the article. Robin Purshouse and Nicholas Latimer provided guidance and expertise. All authors read and approved the final manuscript.

## Conflict of Interest Statement

The authors declare that the research was conducted in the absence of any commercial or financial relationships that could be construed as a potential conflict of interest.

## Supplementary Material

The Supplementary Material for this article can be found online at http://www.frontiersin.org/Journal/10.3389/fpsyt.2014.00114/abstract
