# Supplementary material for: What are the Implications for Policy Makers? A Systematic Review of the Cost-Effectiveness of Screening and Brief Interventions for Alcohol Misuse in Primary Care
Source: Front Psychiatry. 2014 Sep 1;5:114. doi: 10.3389/fpsyt.2014.00114 (PMC4150206; doi:10.3389/fpsyt.2014.00114)
Supplement: Supplementary file 1 [file Presentation1.PDF]

Appendix A – Inclusion/Exclusion criteria

- Studies evaluating a population of adults & young people aged 10+ were included
- Studies reporting screening by use of i) alcohol screening questionnaires, ii) biochemical indicators of alcohol misuse or iii) clinical indicators of alcohol misuse to identify individuals who currently misuse or are at risk of misusing alcohol were included
- Outcomes – studies reporting costs, QALYs and other economic outcomes were included
- Study types: cost-effectiveness, cost utility and cost-consequence studies were included
- Studies not published in English were excluded
- Studies where any aspect of the screening or intervention took place outside of primary care were excluded
- Studies relating to drink-driving, schools, education, pregnancy or self-help interventions or those administered by an alcohol specialist were excluded
- Studies which examine only screening without a brief intervention component were excluded
- Interventions consisting of more than 4 sessions were excluded
- Interventions addressing risk factors other than alcohol (e.g. combined alcohol and drug interventions) were excluded
- Studies addressing SBI implementation strategies (i.e. GP education programmes to increase delivery rates of SBIs to patients) were excluded unless they presented a separate economic evaluation of the SBI delivery.

Appendix B – Data extraction template

- Bibliographic reference
- Study Type
- Population
- Country
- Perspective
- Screening method
- Screening delivery staff
- Intervention (type, frequency & duration)
- Comparator (type, frequency & duration)
- Intervention delivery staff
- Total intervention duration (contact time, excluding screening)
- Source of effectiveness data
- Method of eliciting health valuations
- Costs included
- Currency & year
- Cost/patient per alternative
- Effect/patient per alternative
- ICER
- Approach to handling uncertainty
- Time horizon & discount rate
- Source of funding
- Comments
- Study quality (++,+,-)

Appendix C – Excluded studies

Bibliographic Reference	Reason for exclusion
Clifford A, Shakeshaft A, Deans C. Training and tailored outreach support to improve alcohol screening and brief intervention in Aboriginal Community Controlled Health Services. Drug and Alcohol Review 2013, 32, 72-79	Study examines implementation, not delivery
Kisely S, Asbridge M, Connor J, White A, Pais J, Lin E. Using administrative health data for the surveillance of interventions for alcohol-related harm among young people. Canadian Medical Association Journal 2012, 184(1)	Study does not present an economic evaluation - only discusses data one might use in such an evaluation
Regan D. The brief alcohol intervention. The psychologist 2012, 25(4).	Study presents only a review of existing evidence
Ewing T, Barrios C, Lau C, Patel M, Cui E, Garcia SD, Kong A, Lotfipour S, Lekawa M, Malinoski D. Predictors of hazardous drinking behavior in 1,340 adult trauma patients: a computerized screening and intervention study. Journal of the American College of Surgeons 2012, 215(4)	Not set in primary health care (PHC)
Miller MB, Leffingwell T, Claborn K, Meier E, Walters S, Neighbors C. Personalized feedback interventions for college alcohol misuse: an update of Walters & Neighbors (2005). Psychology of Addictive Behaviors 2012.	Review of studies set in colleges, not PHC
Bray JW, Zarkin GA, Hinde JM, Mills MJ. Costs of alcohol screening and brief intervention in medical settings: a review of the literature. Journal of Studies on Alcohol and Drugs 2012, 73, 911-919	Review of SBI costs only, health outcomes excluded
Cowell AJ, Bray JW, Mills MJ, Hinde JM. Conducting economic evaluations of screening and brief interventions for hazardous drinking: methods and evidence to date for informing policy. Drug and Alcohol Review 2010, 29(6), 623-630	Review of methods for economic evaluation of SBIs, not an evaluation itself
Smit F, Lokkerbol J, Riper H, Majo MC, Boon B, Blankers M. Modeling the cost-effectiveness of health care systems for alcohol use disorders: how implementation of ehealth interventions improves cost-effectiveness. Journal of Medical Internet Research 2011, 13(3), e56	No independent evaluation of SBI presented - study examines either SBI+referral to specialist care or eHealth intervention
Holm AL, Veerman L, Cobiac L, Ekholm O, Diderichsen F. Cost-effectiveness of preventative interventions to reduce alcohol consumption in Denmark. PLoS ONE 2014, 9(2), e88041	SBI option is delivered by telephone, not in primary care
Zarkin GA, Bray JW, Davis KL, Babor TF, Higgins-Biddle JC. The costs of screening and brief intervention for risky alcohol use. Journal of Studies on Alcohol and Drugs 2003, 64(6), 849-857	Study presents only cost outcomes, not health
Shakeshaft AP, Bowman JA, Burrows S, Doran CM, Sanson-Fisher RW. Community-based alcohol counselling: a randomized clinical trial. Addiction 2002, 97(11), 1449-1463	Setting in community counselling centres, not PHC
Downs S, Klein J. Clinical preventive services efficacy and adolescents' risky behaviours. Archives of Pediatrics and Adolescent Medicine 1995, 149(4), 374-379	Intervention is office based, not in primary care
Bradley KA, DeBenedetti AF, Volk RJ, Williams EC, Frank D, Kivlahan DR. AUDIT-C as a brief screen for alcohol misuse in primary care. Alcoholism: Clinical and Experimental Research 2007, 31(7), 1208-1217	Study presents only effectiveness estimates for different screening tools/thresholds, no costs presented
Desai MM, Rosenheck RA, Craig TJ. Screening for alcohol use disorders among medical outpatients: the influence of individual and facility characteristics. American Journal of Psychiatry 2005, 162, 1521-1526	Study presents cost estimates of screening only, not BI
Israel Y, hollander O, Sanchez-Craig M, Booker S, Miller V, Gingrich R, Rankin J. Screening for problem drinking and counselling by the primary care physician-nurse team. Alcoholism: Clinical and Experimental Research 1996, 20(8), 1443-1450	Intervention is too intensive to be considered BI
Andrews G, Issakidis C, Sanderson K, Corry J, Lapsley H. Utilising survey data to inform public policy: comparison of the cost effectiveness of treatment of ten mental disorders. British Journal of Psychiatry 2004, 184, 526-533	Holistic evaluation of cost-effectiveness of 'optimal treatment' vs. current care for patients with Alcohol Use Disorders. Not possible to identify costs and outcomes of SBI components seperately and not clear if this is modelled as delivered in PHC rather than specialist treatment services
Corry J, Sanderson K, Issakidis C, Andrews G, Lapsley H. Evidence-based care for alcohol use disorders is affordable. Journal of Studies on Alcohol 2004	
Broskowski A, Smith S. Estimating the cost of preventive services in mental health and substance abuse under managed care. Rockville, MD: U.S. Department of Health and Human Services, Substance Abuse and Mental Health Services Administration; 2001.	Study estimates costs of SBI only, not outcomes
Mortimer D, Segal L. Economic evalaution of interventions for problem drinking and alcohol dependence: cost per QALY estimates. Alcohol & Alcoholism 2005, 40(6), 549-555	Study models SBIs in a range of settings (not just PHC)
Mortimer D, Segal L. Economic evaluation of interventions for problem drinking and alcohol dependence: do within-family external effects make a difference? Alcohol & Alcoholism 2006, 41(1), 92-98	
Lindholm L. Alcohol advice in primary health care - is it a wise use of resources? Health Policy 1998, 45(1), 47-56	Intervention is too intensive to be considered BI
Coulton S, Drummond C, James D, Godfrey C, Bland JM, Parrott S, Peters T. Opportunistic screening for alcohol use disorders in primary care: comparative study. BMJ 2006, 332(7540), 511-517	Study examines screening only, no BI component

Appendix D – Glossary

Alcohol Use Disorders Identification Test (AUDIT) – A ten-question diagnostic test designed to identify harmful alcohol consumption. The first three questions in isolation are referred to as AUDIT-C.

CAGE – A four question diagnostic test designed to identify alcoholism.

Carbohydrate Deficient Transferrin – A laboratory test used to detect alcohol consumption

Cost-Benefit Analysis (CBA) - An economic evaluation that expresses both costs and outcomes of an intervention in monetary terms.

Cost-Effectiveness Analysis (CEA) - An economic study design in which consequences of different interventions are measured using a single outcome, usually in 'natural' units (for example, life-years gained, deaths avoided, heart attacks avoided, or cases detected).

Cost-Utility Analysis (CUA) - A form of cost-effectiveness analysis in which benefits are measured using a common outcome measure such as the QALY or the DALY.

Disability-Adjusted Life Year (DALY) – A measure of health outcomes equivalent to one year lost of 'healthy' life, incorporating both premature mortality and time lived in less than perfect health.

Dominated – An intervention which is both more costly and with worse health outcomes than the comparator in the analysis

Dominates/dominating – An intervention which is both health-improving and cost-saving with respect to the comparator in the analysis

Economic Evaluation Alongside a Controlled Trial (EEACT) – A study design in which a health economic evaluation is conducted in conjunction with a clinical trial, with observed costs and health outcomes of the trial patients used in the analysis. The time frame of such studies is therefore tied to the duration of the trial

Incremental Cost-Effectiveness Ratio (ICER) – The difference in the mean costs of an intervention compared with the next best alternative divided by the difference in mean health outcomes (usually expressed in terms of cost per QALY or DALY gained).

Life Year Saved (LYS) – A measure of health outcomes equivalent to one additional year of life, without accounting for the health-related quality of life in that year.

Modelling study – A study design in which existing evidence, often from multiple sources, is synthesised into a single mathematical framework in order to estimate the costs and health outcomes of an intervention. Often used to estimate the impacts of an intervention in the longer-term for which no trial data may be available.

Quality-Adjusted Life Year (QALY) – A measure of health outcomes equivalent to one year lived in full health. Similar in concept to the DALY, although QALYs measure health gain, whilst DALYs measure health loss.

SF-12 – A 12-item short-form questionnaire, derived from the longer SF-36, designed to measure the respondent’s health
